# Alcohol vs. Cocaine: Impulsivity and Alexithymia in Substance Use Disorder

**DOI:** 10.3390/bs15060711

**Published:** 2025-05-22

**Authors:** Alessio Mosca, Giovanna Bubbico, Clara Cavallotto, Stefania Chiappini, Rita Allegretti, Andrea Miuli, Carlotta Marrangone, Nicola Ciraselli, Mauro Pettorruso, Giovanni Martinotti

**Affiliations:** 1 Department of Neuroscience, Imaging and Clinical Sciences, “G. d’Annunzio” University of Chieti-Pescara, 66100 Chieti, Italymauro.pettorruso@hotmail.it (M.P.); giovanni.martinotti@gmail.com (G.M.); 2 ITAB|Institute for Advanced Biomedical Technologies, 66100 Chieti, Italy; 3 Department of Human and Clinical Sciences, UniCamillus—International Medical University, Via di Sant’Alessandro 8, 00131 Rome, Italy

**Keywords:** substance use disorder, alcohol use disorder, cocaine use disorder, alexithymia, impulsivity, emotional dysregulation, poly-substance use, addiction psychology, emotion regulation

## Abstract

Substance Use Disorders (SUDs) are frequently associated with impairments in emotional regulation and behavioural control. Among the most prevalent substances of abuse, alcohol and cocaine are known to exert distinct effects on neuropsychological functioning. This study aimed to compare individuals with Alcohol Use Disorder (AUD) and Cocaine Use Disorder (CUD) in terms of impulsivity and alexithymia, and to examine the clinical implications of poly-substance use involving both alcohol and cocaine. Participants completed standardized psychometric assessments, including the Barratt Impulsiveness Scale (BIS-11), the Brief Psychiatric Rating Scale (BPRS), and the Toronto Alexithymia Scale (TAS-20). Group comparisons were conducted using non-parametric tests, and logistic regression models were applied to control for demographic covariates. The findings showed that impulsivity levels were comparable across groups, whereas alexithymia scores were significantly higher in individuals with AUD and in those with poly-substance use, relative to CUD-only participants. These findings underscore the relevance of targeting emotional regulation difficulties, particularly alexithymia, in the assessment and treatment of SUDs. Integrating emotion-focused interventions may enhance treatment outcomes, especially for individuals with co-occurring substance use patterns. Future research is needed to clarify the underlying neuropsychological mechanisms contributing to these differences and to inform more personalized approaches to addiction care.

## 1. Introduction

Substance Use Disorders (SUDs), including Alcohol Use Disorder (AUD) and Cocaine Use Disorder (CUD), represent major global public health concerns, significantly impacting individuals’ physical, psychological, and social well-being. In Europe alone, an estimated 66.2 million people aged 15 and older are affected by AUD (World Health Organization, 2018), resulting in considerable economic and societal burdens, as well as increased risk of comorbid conditions such as cardiovascular disease, metabolic disorders, and premature mortality (Zizzi et al., 2024). Although less prevalent, approximately 22 million people worldwide used cocaine in 2021 (United Nations Office on Drugs and Crime, 2023). Cocaine use remains among the most addictive and difficult-to-treat forms of SUD, with a recent increase in cocaine purity (EMCDDA, 2022) contributing to high relapse rates and frequent psychiatric comorbidities, including anxiety and depression (Hallgren & Witkiewitz, 2015). While alcohol and cocaine differ pharmacologically, both substances exert a profound influence on dopaminergic pathways in the brain, contributing to maladaptive reward processing and impaired impulse control (Boden & Fergusson, 2011; Giustiniani et al., 2022).

A growing body of research highlights the relevance of specific personality traits in the onset, maintenance, and treatment outcomes of SUDs (Zilberman et al., 2020). Among these, alexithymia—the difficulty in identifying and describing one’s emotions—and impulsivity—the tendency to act without adequate forethought—are consistently linked to higher vulnerability for substance misuse and poorer treatment outcomes (Zizzi et al., 2024; Ziółkowski et al., 1995). Alexithymia is particularly prevalent in individuals with AUD, where it is associated with elevated levels of depression, impulsivity, hopelessness and lack of empaty (Martinotti et al., 2009), all of which may contribute to increased relapse risk and reduced therapeutic engagement (Palma-Álvarez et al., 2021). Similarly, in CUD, alexithymia is linked to emotional dysregulation and difficulty maintaining abstinence, complicating long-term recovery efforts (Achab et al., 2013). Evidence suggests that individuals with alexithymia are more prone to using substances as a maladaptive coping strategy, perpetuating a vicious cycle of dependency and emotional dysfunction (Taylor et al., 1997; Craparo et al., 2014).

Impulsivity has likewise been identified as a key factor in the trajectory and severity of both AUD and CUD (Dalley & Ersche, 2019). In AUD, high impulsivity correlates with earlier relapse and challenges in adhering to treatment regimens (Loree et al., 2015). In CUD, impulsivity is strongly associated with early onset of drug use, increased craving, and more severe addiction profiles (Urueña-Méndez et al., 2023; Leeman & Potenza, 2014; Mosca et al., 2023). Notably, impulsivity and alexithymia often co-occur and may interact synergistically: individuals with high levels of both traits tend to exhibit more profound emotional dysregulation, resulting in more complex clinical presentations and reduced treatment responsiveness (Gori et al., 2014; Pepe et al., 2023).

Both AUD and CUD are frequently complicated by psychiatric comorbidities, particularly depression and anxiety, which may serve as both precursors and consequences of substance use (McHugh & Weiss, 2019; Obeid et al., 2021). In individuals with AUD, depressive symptoms are associated with greater risk of relapse and poorer long-term outcomes, underscoring the importance of integrated treatment approaches that address both substance use and co-occurring psychopathology (Boden & Fergusson, 2011; Hallgren & Witkiewitz, 2015). The combination of alexithymia, impulsivity, and mood disturbances such as hopelessness and suicidality further compound this clinical complexity, increasing the need for tailored and multidisciplinary interventions (Sadock & Sadock, 2009; Stoffers et al., 2010).

Given these interrelated risk factors, a better understanding of the roles of alexithymia and impulsivity across different SUD profiles—particularly in individuals with single-substance (AUD or CUD) and poly-substance use—may help identify psychological targets for more personalized interventions. The present study aims to examine differences in alexithymia, impulsivity, and psychiatric symptom severity across individuals with AUD, CUD, and the combined use of both substances. By identifying specific emotional and cognitive traits associated with each group, this research seeks to inform more effective, individualized treatment strategies and support sustained recovery (Craparo et al., 2014; Boden & Fergusson, 2011). We hypothesized that individuals with AUD would exhibit higher levels of alexithymia and psychiatric symptom severity compared to those with CUD, while impulsivity would be greater in individuals with CUD and highest among those with poly-substance use.

## 2. Materials and Methods

### 2.1. Participants and Sample Characteristics

This study included a total of 119 participants, comprising 39 individuals with a diagnosis of Alcohol Use Disorder (AUD) and 80 individuals with a primary diagnosis of Cocaine Use Disorder (CUD). Among individuals with a primary diagnosis of CUD, 10 reported exclusive cocaine use, while 70 engaged in polydrug use (including alcohol, cannabis, and opioids). In contrast, individuals diagnosed with AUD did not exhibit patterns of polydrug use. Participants were recruited from various mental health institutions in Italy, including the Psychiatric Diagnostic and Treatment Service of the S.S. Annunziata University Hospital in Chieti and the Inpatient Psychiatric Center of Villa Maria Pia in Rome. All diagnoses were made according to standardized criteria for Substance Use Disorders (SUDs), in accordance with internationally recognized diagnostic systems.

All participants provided written informed consent prior to their inclusion in the study. The protocol was approved by the institutional ethics committee and conducted in accordance with the Declaration of Helsinki.

Demographic information collected included age, sex, years of education, employment status (coded as 1 = employed, 0 = unemployed), and marital status (recoded into a binary variable: 1 = married or partnered, 0 = single). Descriptive statistics for all variables were calculated separately for the AUD and CUD groups.

### 2.2. Psychometric Evaluation

Participants were evaluated using three validated psychometric instruments. Emotional regulation difficulties were measured using the 20-item Toronto Alexithymia Scale (TAS-20), which evaluates three dimensions: difficulty identifying feelings, difficulty describing feelings, and externally oriented thinking. The total score ranges from 20 to 100, with higher scores indicating greater alexithymia.

Impulsivity was assessed using the Barratt Impulsiveness Scale (BIS-11), a 30-item self-report instrument that covers attentional, motor, and non-planning impulsivity. Each item is rated on a 4-point Likert scale, and higher total scores reflect greater levels of impulsivity.

Psychiatric symptom severity was measured using the Brief Psychiatric Rating Scale (BPRS), a clinician-administered instrument that evaluates a range of symptoms such as depression, anxiety, and hostility across 18 items rated on a 7-point scale. These instruments are widely used in both clinical and research contexts. The Toronto Alexithymia Scale (TAS-20) has demonstrated good internal consistency, with Cronbach’s alpha values ranging from 0.75 to 0.82 (Bagby et al., 1994; Bressi et al., 1996). The Italian version of the BIS-11 has shown acceptable reliability, with a Cronbach’s alpha of 0.79 (Fossati et al., 2001). The Brief Psychiatric Rating Scale (BPRS) also exhibits robust inter-rater reliability and internal consistency across studies (Ventura et al., 1993).

## 3. Results

### 3.1. Demographic and Clinical Differences

The CUD group had a higher proportion of males (90%) compared to the AUD group (25.6%). Participants in the AUD group were older (mean age = 52.41 years, SD = 10.20) than those in the CUD group (mean age = 38.36 years, SD = 7.82). Years of education were comparable between groups, with means of 11.95 (SD = 3.84) in the AUD group and 12.65 (SD = 3.69) in the CUD group. Employment status differed, with 70% of CUD participants being employed, compared to 38.5% in the AUD group. Marital status also varied, with AUD participants reporting higher mean scores for being married or in a partnership (mean = 1.08, SD = 0.90) than CUD participants (mean = 0.65, SD = 0.60). Chi-square analyses revealed significant differences across several demographic and clinical variables. The AUD group showed a higher prevalence of dual diagnosis (*p* < 0.001), and a greater use of antidepressants and mood stabilizers (*p* < 0.001). In contrast, cannabis use was more common in the CUD group (*p* < 0.001). No significant differences were found between groups regarding opioid use (*p* = 0.11). See Table 1.

### 3.2. Psychological and Clinical Measures

**Figure 1:**
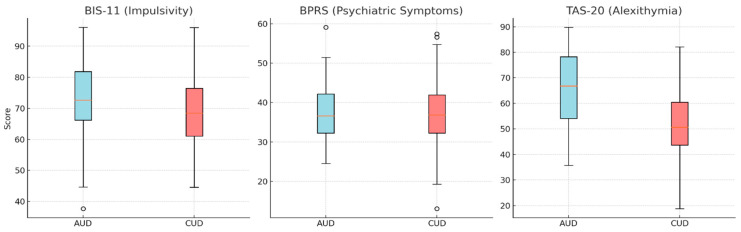
Group comparisons of psychological measures between individuals with Alcohol Use Disorder (AUD) and Cocaine Use Disorder (CUD). Boxplots display the distribution of scores on the BIS-11 (Impulsivity), BPRS (Psychiatric Symptoms), and TAS-20 (Alexithymia). No significant difference was observed in impulsivity scores between groups (*p* = 0.97). A trend toward higher psychiatric symptom severity was found in the AUD group (*p* = 0.056), while alexithymia scores were significantly higher in the AUD group compared to the CUD group (*p* = 0.012), suggesting greater emotional dysregulation among individuals with alcohol dependence.

Mann–Whitney U tests were conducted to compare psychological characteristics between the AUD and CUD groups. No significant differences were observed in impulsivity scores as measured by the BIS-11 (*p* = 0.97), indicating comparable levels of impulsivity across the two groups. Psychiatric symptom severity, assessed using the BPRS, was higher in the AUD group; however, this difference did not reach statistical significance (*p* = 0.056). In contrast, alexithymia scores, as measured by the TAS-20, were significantly higher in the AUD group compared to the CUD group (*p* = 0.012), suggesting greater difficulties in emotional regulation among individuals with alcohol dependence (See Table 2). These findings are visually presented in Figure 1.

### 3.3. CUD-Only vs. Poly-Substance Use Comparison

A separate comparison was conducted between individuals with Cocaine Use Disorder only (CUD-only) and those with poly-substance use involving both cocaine and alcohol. Impulsivity scores, as measured by the BIS-11, did not differ significantly between the two groups (*p* = 0.648), indicating comparable levels of impulsivity. In contrast, psychiatric symptom severity (BPRS) was significantly higher in the poly-use group (*p* = 0.034), suggesting greater psychiatric distress. Similarly, alexithymia scores (TAS-20) were significantly elevated in the poly-use group compared to the CUD-only group (*p* = 0.048), indicating increased difficulty in emotional processing and regulation. These findings are visually summarized in Figure 1, which highlights the elevated BPRS and TAS-20 scores among poly-substance users, despite stable impulsivity levels across groups.

### 3.4. Regression Analysis

To identify psychological and demographic predictors of poly-substance use (defined as concurrent use of alcohol and cocaine) versus cocaine-only use, a binary logistic regression model was conducted. The predictors included age, years of education, impulsivity (BIS-11), psychiatric symptom severity (BPRS), and alexithymia (TAS-20).

The logistic regression model was statistically significant (Likelihood Ratio χ^2^ = 10.80, *p* = 0.056), with a pseudo-R^2^ of 0.107, indicating that approximately 10.7% of the variance in poly-substance use status was explained by the included predictors.

Among the variables tested, alexithymia (TAS-20) emerged as the strongest predictor, approaching statistical significance (β = 0.0447, *p* = 0.0698). This suggests that greater emotional dysregulation may be associated with an increased likelihood of poly-substance use. Psychiatric symptoms (BPRS) also showed a trend toward significance (*p* = 0.1061), while impulsivity (BIS-11), age, and education were not significant predictors (*p* > 0.30 for all). See Table 3.

These findings support the potential role of alexithymia as a key psychological trait in distinguishing more complex addiction profiles.

## 4. Discussion

To the best of our knowledge, this is the first study comparing impulsivity, psychiatric symptom severity, and alexithymia between individuals with Alcohol Use Disorder (AUD) and those with Cocaine Use Disorder (CUD). Furthermore, we explored differences between CUD-only individuals and those engaged in poly-substance use (cocaine and alcohol). Our findings offer valuable insights into the emotional and cognitive profiles associated with different substance use patterns.

First, we observed that alexithymia levels were significantly higher in the AUD group compared to those with CUD, consistent with prior research highlighting alexithymia as a core feature in alcohol dependence (Thorberg et al., 2009; Evren et al., 2008, 2012). This may reflect a stronger tendency among individuals with AUD to use alcohol as a maladaptive strategy for regulating emotional discomfort, particularly when emotional awareness and articulation are impaired. In contrast, individuals with CUD showed relatively lower levels of alexithymia, possibly indicating different emotional or motivational dynamics underlying cocaine use.

From another perspective, numerous scientific studies have demonstrated that chronic alcohol use can alter brain regions involved in emotional regulation and processing, such as the insula (Centanni et al., 2020), amygdala (Pandey et al., 2008), and prefrontal cortex (Crews et al., 2016), suggesting that higher rates of alexithymia may be more closely associated with the neurobiological effects of chronic alcohol consumption rather than with self-medication attempts.

Although impulsivity is widely documented in both AUD and CUD populations (Barratt, 1985; Gori et al., 2014), our data did not reveal significant group differences on the BIS-11. This suggests that impulsivity may be a transdiagnostic trait across SUDs, without strong specificity to alcohol or cocaine. However, this finding should be interpreted with caution, as BIS-11 may not fully capture the nuanced, multidimensional nature of impulsivity across substances.

A trend toward higher psychiatric symptom severity (BPRS) was observed in the AUD group, though it did not reach statistical significance. This aligns with the literature suggesting that alcohol dependence is frequently associated with mood disorders and affective dysregulation (Hallgren & Witkiewitz, 2015), which may compound emotional processing difficulties such as alexithymia (Evren et al., 2008, 2012). In contrast, psychiatric symptoms in CUD may be more state-dependent, linked to acute intoxication or withdrawal phases.

When examining poly-substance users (Cocaine + Alcohol) versus CUD-only individuals, we found that the poly-use group exhibited significantly higher psychiatric symptoms and alexithymia scores, despite no difference in impulsivity. This pattern supports previous research suggesting that individuals who use multiple substances may represent a more clinically severe subgroup, characterized by emotional dysregulation, comorbid psychopathology, and poorer functional outcomes (Craparo et al., 2014; Zizzi et al., 2024). The finding that alexithymia emerged as a marginally significant predictor of poly-substance use in the regression analysis further reinforces its potential role as a psychological marker of complexity and treatment resistance in addiction.

These results have important clinical implications. First, they highlight the need to assess alexithymia routinely in substance use treatment settings, particularly for individuals with alcohol dependence or co-occurring substance use. In light of this, a psychotherapeutic intervention specifically targeting alexithymic traits could be therapeutic in individuals with Substance Use Disorder (SUD) (Saeedi Rashkolia et al., 2022; Zargar et al., 2019). From a pharmacological standpoint, mood stabilizers may be effective by improving alexithymic traits (Chaim et al., 2024; Brown et al., 2018), whereas antipsychotics should be avoided unless a clear comorbidity is present (Lysaker et al., 2005; Van’t Wout et al., 2007). Second, the absence of group differences in impulsivity emphasizes the importance of tailoring interventions beyond trait impulsivity and focusing more deeply on emotion processing skills.

Limitations of the present study include its cross-sectional design, which precludes causal inference, and the reliance on self-report measures, which may be subject to social desirability or insight limitations, particularly in individuals with alexithymia. Additionally, the regression model encountered quasi-complete separation, limiting interpretation of some predictors due to statistical instability. A larger and more balanced sample, particularly in terms of gender, would strengthen generalizability.

## 5. Conclusions

This study reveals meaningful differences in the clinical and psychological profiles of individuals with Alcohol Use Disorder (AUD) and Cocaine Use Disorder (CUD). Our findings highlight alexithymia as a key psychological trait particularly associated with AUD and poly-substance use, suggesting its potential as a clinical marker of emotional dysregulation in addiction.

Future research should examine the longitudinal course and treatment outcomes linked to alexithymia, ideally through integrated neurobiological and behavioural frameworks to better understand its mechanistic role in Substance Use Disorders (SUDs). Additionally, studies should investigate the neurobiological underpinnings of emotional dysregulation in addiction, employing techniques such as functional neuroimaging to identify distinct patterns of brain activity associated with alexithymia across different substance use profiles.

Exploring how demographic variables—such as sex, age, and employment status—interact with psychological traits like impulsivity and alexithymia may further clarify the heterogeneity of addiction pathways, supporting the development of personalized, precision-based therapeutic interventions.

## Figures and Tables

**Table 1:** Participants.

	Sex (Alc)	Sex (Coca)	Age (Alc)	Age (Coca)	Educyears (Alc)	Educyears (Coca)	Employed (Alc)	Employed (Coca)	Marital Status (Alc)	Marital Status (Coca)
Valid	39.0	80.00	39.00	80.00	39.000	80.000	39.000	80.000	39.00	80.00
Mean	0.25	0.900	52.41	38.36	11.949	12.650	0.385	0.700	1.077	0.650
Std. Deviation	0.44	0.302	10.20	7.818	3.839	3.691	0.493	0.461	0.900	0.597
Minimum	0.00	0.000	30.0	19.00	3.000	0.000	0.000	0.000	0.000	0.000
Maximum	1.00	1.000	74.0	57.00	18.000	25.000	1.000	1.000	3.000	2.000

**Table 2:** Descriptive Statistics for Psychological Measures by Group. Descriptive statistics for impulsivity (BIS-11), psychiatric symptoms (BPRS), and alexithymia (TAS-20) for individuals with Alcohol Use Disorder (AUD) and Cocaine Use Disorder (CUD). For each measure, mean, standard deviation, minimum, and maximum values are reported.

	BIS-11 (Alc)	BIS-11 (Coca)	BPRS (Alc)	BPRS (Coca)	TAS-20 (Alc)	TAS-20 (Coca)
Valid	39	80	39	80	39	80
Mean	68.590	69.400	40.744	37.513	59.615	52.737
SD	12.115	11.170	9.397	8.812	15.508	12.758
Minimum	45.000	37.000	24.000	20.000	21.000	25.000
Maximum	92.000	92.000	61.000	59.000	92.000	89.000

**Table 3:** Logistic Regression Predicting Poly-Substance Use (Alcohol + Cocaine vs. Cocaine Only) Binary logistic regression results examining the effects of age, education, impulsivity (BIS-11), psychiatric symptom severity (BPRS), and alexithymia (TAS-20) on the likelihood of poly-substance use.

Predictor	β (Coef.)	Std. Error	z-Value	*p*-Value	95% CI (Lower)	95% CI (Upper)
Age	0.025	0.028	0.909	0.364	−0.029	0.079
Education (years)	0.075	0.074	1.014	0.312	−0.070	0.219
BIS-11 (impulsivity)	−0.019	0.029	−0.669	0.505	−0.075	0.037
BPRS (Psych. Syntoms)	0.062	0.038	1.616	0.106	−0.013	0.137
TAS-20 (Alexithymia)	0.045	0.025	1.814	0.070	−0.004	0.093

## Data Availability

The original contributions presented in this study are included in the article. Further inquiries can be directed to the corresponding author.

## References

[B1-behavsci-15-00711] Achab S., Nicolier M., Masse C., Vandel P., Bennabi D., Mauny F., Haffen E. (2013). Alexithymia and impulsivity as common features in addictive disorders. Journal of Addiction Research & Therapy.

[B2-behavsci-15-00711] Bagby R. M., Parker J. D. A., Taylor G. J. (1994). The twenty-item Toronto Alexithymia Scale: I. Item selection and cross-validation of the factor structure. Journal of Psychosomatic Research.

[B3-behavsci-15-00711] Barratt E. S., Spence J. T., Izard C. E. (1985). Impulsiveness subtraits: Arousal and information processing. Motivation, emotion, and personality.

[B4-behavsci-15-00711] Boden J. M., Fergusson D. M. (2011). Alcohol and depression: Causation, mechanisms, and treatment. Alcohol Research & Health.

[B5-behavsci-15-00711] Bressi C., Taylor G., Parker J., Bressi S., Brambilla V., Aguglia E., Allegranti I., Bongiorno A., Giberti F., Bucca M., Todarello O., Callegari C., Vender S., Gala C., Invernizzi G. (1996). Cross validation of the factor structure of the 20-item Toronto Alexithymia Scale: An Italian multicenter study. Journal of Psychosomatic Research.

[B6-behavsci-15-00711] Brown T. A., Avery J. C., Jones M. D., Anderson L. K., Wierenga C. E., Kaye W. H. (2018). The impact of alexithymia on emotion dysregulation in anorexia nervosa and bulimia nervosa over time. European Eating Disorders Review.

[B7-behavsci-15-00711] Centanni S. W., Morris B. D., Luchsinger J. R., Bedse G., Fetterly T. L., Patel S., Winder D. G. (2020). Endocannabinoid control of the insular–amygdalar circuit regulates alcohol intake. Frontiers in Behavioral Neuroscience.

[B8-behavsci-15-00711] Chaim C. H., Almeida T. M., de Vries Albertin P., Santana G. L., Siu E. R., Andrade L. H. (2024). The implication of alexithymia in personality disorders: A systematic review. BMC Psychiatry.

[B9-behavsci-15-00711] Craparo G., Gori A., Iraci Sareri G., Caretti V. (2014). Antisocial and psychopathic personalities in a sample of addicted subjects: Differences in psychological resources, symptoms, alexithymia, and impulsivity. Comprehensive Psychiatry.

[B10-behavsci-15-00711] Crews F. T., Vetreno R. P., Broadwater M. A., Robinson D. L. (2016). Adolescence, alcohol, and the developing brain: Insights from studies in humans and animal models. Alcohol Research: Current Reviews.

[B11-behavsci-15-00711] Dalley J. W., Ersche K. D. (2019). Neural circuitry and mechanisms of waiting impulsivity: Relevance to addiction. Philosophical Transactions of the Royal Society B-Biological Sciences.

[B12-behavsci-15-00711] EMCDDA (2022). EMCDDA operating guidelines for the European Union Early Warning System on new psychoactive substances.

[B13-behavsci-15-00711] Evren C., Cınar O., Evren B., Celik S. (2012). Relationship between defense styles, alexithymia, and personality in alcohol-dependent inpatients. Comprehensive Psychiatry.

[B14-behavsci-15-00711] Evren C., Evren B., Dalbudak E., Cakmak D. (2008). Alexithymia and personality in relation to social anxiety among male alcohol-dependent inpatients. Archives of Neuropsychiatry.

[B15-behavsci-15-00711] Fossati A., Di Ceglie A., Acquarini E., Barratt E. S. (2001). Psychometric properties of an Italian version of the Barratt Impulsiveness Scale-11 (BIS-11) in nonclinical subjects. Journal of Clinical Psychology.

[B16-behavsci-15-00711] Giustiniani J., Nicolier M., Pascard M., Masse C., Vandel P., Bennabi D., Achab S., Mauny F., Haffen E. (2022). Do individuals with internet gaming disorders share personality traits with substance-dependent individuals?. International Journal of Environmental Research and Public Health.

[B17-behavsci-15-00711] Gori A., Craparo G., Caretti V. (2014). Psychological characteristics of addicts with psychopathic and antisocial tendencies. Comprehensive Psychiatry.

[B18-behavsci-15-00711] Hallgren K. A., Witkiewitz K. (2015). Depression as a mediator of alcohol treatment outcomes. Psychology of Addictive Behaviors.

[B19-behavsci-15-00711] Leeman R. F., Potenza M. N. (2014). Similarities and differences between pathological gambling and substance use disorders: A focus on impulsivity and compulsivity. Psychopharmacology.

[B20-behavsci-15-00711] Loree A. M., Lundahl L. H., Ledgerwood D. M. (2015). Impulsivity as a predictor of treatment outcome in substance use disorders: Review and synthesis. Drug and Alcohol Review.

[B21-behavsci-15-00711] Lysaker P. H., Davis L. W., Hunter N. L., Nees M. A., Wickett A. (2005). Alexithymia and schizophrenia: Associations with symptoms, neurocognition, and social function. Psychiatry Research.

[B22-behavsci-15-00711] Martinotti G., Di Nicola M., Tedeschi D., Cundari S., Janiri L. (2009). Empathy ability is impaired in alcohol-dependent patients. The American Journal on Addictions.

[B23-behavsci-15-00711] McHugh R. K., Weiss R. D. (2019). Alcohol use disorder and depressive disorders. Alcohol Research: Current Reviews.

[B24-behavsci-15-00711] Mosca A., Miuli A., Mancusi G., Chiappini S., Stigliano G., De Pasquale A., Di Petta G., Bubbico G., Pasino A., Pettorruso M., Martinotti G. (2023). To bridge or not to bridge: Moral judgement in cocaine use disorders, a case-control study on human morality. Social Neuroscience.

[B25-behavsci-15-00711] Obeid S., Haddad C., Fares K., Malaeb D., Sacre H., Akel M., Salameh P., Hallit S. (2021). Correlates of emotional intelligence among Lebanese adults: The role of depression, anxiety, suicidal ideation, alcohol use disorder, alexithymia and work fatigue. BMC Psychology.

[B26-behavsci-15-00711] Palma-Álvarez R. F., Ros-Cucurull E., Daigre C., Perea-Ortueta M., Serrano-Pérez P., Martínez-Luna N., Salas-Martínez A., Robles-Martínez M., Ramos-Quiroga J. A., Roncero C., Grau-López L. (2021). Alexithymia in patients with substance use disorders and its relationship with psychiatric comorbidities and health-related quality of life. Frontiers in Psychiatry.

[B27-behavsci-15-00711] Pandey S. C., Ugale R., Zhang H., Tang L., Prakash A. (2008). Brain chromatin remodeling: A novel mechanism of alcoholism. Journal of Neuroscience.

[B28-behavsci-15-00711] Pepe M., Di Nicola M., Panaccione I., Franza R., De Berardis D., Cibin M., Janiri L., Sani G. (2023). Impulsivity and alexithymia predict early versus subsequent relapse in patients with alcohol use disorder: A 1-year longitudinal study. Drug and Alcohol Review.

[B29-behavsci-15-00711] Sadock B. J., Sadock V. A. (2009). Kaplan and Sadock’s synopsis of psychiatry: Behavioral sciences/clinical psychiatry.

[B30-behavsci-15-00711] Saeedi Rashkolia A., Manzari Tavakoli A., Zeinaddiny Meymand Z., Hosseini Fard S. M. (2022). The effectiveness of interpersonal psychotherapy on alexithymia, emotion regulation, and psychological capital of male substance abusers treated by addiction treatment centers in Kerman. Addict Health.

[B31-behavsci-15-00711] Stoffers J., Völlm B. A., Rücker G., Timmer A., Huband N., Lieb K. (2010). Pharmacological interventions for borderline personality disorder. The Cochrane Database of Systematic Reviews.

[B32-behavsci-15-00711] Taylor G. J., Bagby R. M., Parker J. D. (1997). Disorders of affect regulation: Alexithymia in medical and psychiatric illness.

[B33-behavsci-15-00711] Thorberg F. A., Young R. M., Sullivan K. A., Lyvers M. (2009). Alexithymia and alcohol use disorders: A critical review. Addictive Behaviors.

[B34-behavsci-15-00711] United Nations Office on Drugs and Crime (2023). World Drug Report 2023.

[B35-behavsci-15-00711] Urueña-Méndez G., Dimiziani A., Belles L., Goutaudier R., Ginovart N. (2023). Repeated cocaine intake differentially impacts striatal D(2/3) receptor availability, psychostimulant-induced dopamine release, and trait behavioral markers of drug abuse. International Journal of Molecular Sciences.

[B36-behavsci-15-00711] Van’t Wout M., Aleman A., Bermond B., Kahn R. S. (2007). No words for feelings: Alexithymia in schizophrenia patients and first-degree relatives. Comprehensive Psychiatry.

[B37-behavsci-15-00711] Ventura J., Green M. F., Shaner A., Liberman R. P. (1993). Training and quality assurance with the brief psychiatric rating scale: “The drift busters”. International Journal of Methods in Psychiatric Research.

[B38-behavsci-15-00711] World Health Organization (2018). Global status report on alcohol and health 2018.

[B39-behavsci-15-00711] Zargar F., Bagheri N., Tarrahi M. J., Salehi M. (2019). Effectiveness of emotion regulation group therapy on craving, emotion problems, and marital satisfaction in patients with substance use disorders: A randomized clinical trial. Iranian Journal of Psychiatry.

[B40-behavsci-15-00711] Zilberman N., Yadid G., Efrati Y., Rassovsky Y. (2020). Who becomes addicted and to what? psychosocial predictors of substance and behavioral addictive disorders. Psychiatry Research.

[B41-behavsci-15-00711] Ziółkowski M., Gruss T., Rybakowski J. K. (1995). Does alexithymia in male alcoholics constitute a negative factor for maintaining abstinence?. Psychotherapy and Psychosomatics.

[B42-behavsci-15-00711] Zizzi A., Berri I. M., Occhipinti M., Escelsior A., Serafini G. (2024). Psychological dimensions in alcohol use disorder: Comparing active drinkers and abstinent patients. Frontiers in Psychiatry.

